# Deep Learning Models for Classification of Deciduous and Permanent Teeth From Digital Panoramic Images

**DOI:** 10.7759/cureus.49937

**Published:** 2023-12-04

**Authors:** Manoj Jaiswal, Megha Sharma, Padmavati Khandnor, Ashima Goyal, Rajendra Belokar, Sandeep Harit, Tamanna Sood, Kanav Goyal, Pallavi Dua

**Affiliations:** 1 Pedodontics and Preventive Dentistry, Postgraduate Institute of Medical Education and Research, Chandigarh, IND; 2 Computer Science and Engineering, Punjab Engineering College, Chandigarh, IND; 3 Production and Industrial Engineering, Punjab Engineering College, Chandigarh, IND; 4 Mechanical Engineering, Punjab Engineering College, Chandigarh, IND

**Keywords:** alexnet, resnet, yolo, teeth detection, panoramic radiograph, deep learning, artificial intelligence

## Abstract

Introduction: Dental radiographs are essential in the diagnostic process in dentistry. They serve various purposes, including determining age, analyzing patterns of tooth eruption/shedding, and treatment planning and prognosis. The emergence of digital radiography technology has piqued interest in using artificial intelligence systems to assist and guide dental professionals. These cutting-edge technologies assist in streamlining decision-making processes by enabling entity classification and localization tasks. With the integration of artificial Intelligence algorithms tailored for pediatric dentistry applications and utilizing automated tools, there is an optimistic outlook on improving diagnostic capabilities while reducing stress and fatigue among clinicians.

Methodology: The dataset comprised 620 images (mixed dentition: 314, permanent dentition: 306). Panoramic radiographs taken were within the age range of 4-16 years. The classification of deciduous and permanent teeth involved training CNN-based models using different architectures such as Resnet, AlexNet, and EfficientNet, among others. A ratio of 70:15:15 was utilized for training, validation, and testing, respectively.

Result and conclusion: The findings indicated that among the models proposed, EfficientNetB0 and EfficientNetB3 exhibited superior performance. Both EfficientNetB0 and EfficientNetB3 achieved an accuracy rate, precision, recall, and F1 scores of 98% in classifying teeth as either deciduous or permanent. This implies that these models were highly accurate in identifying patterns/features within the dataset used for evaluation.

## Introduction

“Pediatric dentistry is a branch of dentistry that deals with the oral healthcare of children from birth through to adolescence” [1]. One of the integral components of this specialty involves studying eruption and shedding sequence and crown and root completion throughout the various stages of calcification/maturation of teeth, along with age estimation through clinical and radiographic examinations.

Variations in the eruption sequence of deciduous, mixed, and permanent dentition can be observed among different ethnic groups. Disruptions in tooth eruption, whether premature or delayed, are often attributed to systemic or environmental factors. In some cases, this may result in the emergence of natal teeth or neonatal teeth. To evaluate such conditions, radiographs prove helpful as they provide a clear perception of normal, primary failure of eruption, ectopically erupted, and impacted teeth [2].

Panoramic radiographs have found extensive use within the field of dentistry due to their numerous advantages. They offer an overall anatomical view encompassing dental arches along with essential structures like temporomandibular joints, maxillary sinuses, and even the hyoid bone. Furthermore, panoramic radiography is relatively simple to perform on patients with limited mouth opening [3].

Dentists undergo extensive training to analyze orthopantomograms (OPGs), but there are various factors that can affect their interpretation. These factors encompass both congenital and acquired anomalies of teeth, such as supernumerary teeth (e.g., mesiodens, peg lateral, paramolars, distomolars), as well as variations in the tooth size and shape. Furthermore, the absence or impaction of a tooth and the proximity of neighboring dental structures may introduce ambiguity in interpretations. As a result, these challenges pose limitations on clinical service efficiency and daily practice workflow within the field.

Regardless of their specific diagnostic ability, all radiographs are prone to inter- and intra-examiner reliability. The utilization of automated assistance systems in the analysis of dental radiographic images holds promise for clinicians in mitigating inconsistencies between examiners.

By implementing such automated tools, the limitations associated with intra- and inter-examiner variability can be addressed effectively. As a result, dentists are provided with objective assessments that not only support their clinical decisions but also contribute toward improving efficiency by reducing the time required for radiograph interpretation [4,5].

There are several techniques available for detecting teeth, some of which rely on pre-trained models while others use customized neural networks that learn from OPG images [6]. However, there is currently a dearth of data regarding the evaluation of radiographic algorithms for mixed dentition using artificial intelligence. Given the numerous advantages that panoramic radiography offers in dentistry, it is crucial for dental clinicians to harness the capabilities of artificial intelligence in order to develop automatic diagnostic models.

The rationale for this study is founded on the critical role of dental radiographs, particularly panoramic images, in pediatric dentistry. The study aims to improve upon traditional radiograph interpretation by utilizing advanced technology, such as deep learning models, to enhance the accuracy and efficiency of tooth classification from panoramic images.

The information garnered from these AI models can play a pivotal role in predicting growth and development of dentitions and associated anomalies. Early diagnosis has significant implications for treatment planning and patient care. As such, this study seeks to examine not only the technical feasibility but also expert-level performance and real-world clinical effectiveness of AI-based tooth recognition through panoramic radiographs.

## Materials and methods

A cross-sectional study was designed to examine OPG images obtained from the Oral Health Sciences Centre at PGIMER Chandigarh. Patient positioning in the OPG machine was performed according to the instructions provided by the manufacturer.

Ethical clearance

Ethical clearance was obtained from the Institute Ethics Committee no: IEC-12/2021-2199 and for the present study, collaborative clearance was obtained from the institutional collaborative research committee to collaborate with Punjab Engineering College, Chandigarh. 

Statistical analysis

In order to classify deciduous and permanent teeth from digital panoramic images, several deep learning models were analyzed. This was done by training different models and evaluating their performance based on various parameters. By calculating these results, we gain a better understanding of how well each model performs in this classification task. The parameters described in the subsequent paragraphs are utilized to define and elucidate the performance of each model.

The accuracy of a prediction is determined by calculating the number of correct predictions across all datasets.

$accuracy = \frac{(True Positive + False Negative)}{(True Positive + True Negative + False Positive + False Negative)}$

Precision, on the other hand, assesses the correctness of positive predictions made.

$precision = \frac{(True Positive )}{(True Positive + False Positive )}$

Recall evaluates how many positive cases were correctly predicted out of all available positive cases in the data.

$recall = \frac{(True Positive )}{(True Positive + False Negative )}$

The F1 score represents the harmonic mean value derived from both precision and recall measurements.

$F1 score = \frac{(2* precision*recall )}{(precision+recall )}$

Dataset description

To preserve patient privacy, all data were anonymized and encrypted/filtered, ensuring that no demographic information such as name, gender, or age could be identified. Initially, the dataset consisted of 1,293 OPG images. However, a thorough evaluation was conducted using inclusion and exclusion criteria to ensure that only relevant images were included in the final analysis. As a result, a total of 620 images meeting specific eligibility criteria were manually selected for further examination. Afterward, these selected images were further divided into training, testing, and validation to get more stable results. Children aged 4 to 16 were included in the study as they are more cooperative with undergoing radiographic procedures. This also allows for a broader range of training data for the AI algorithm, encompassing various dental development stages beyond just mixed or permanent dentition. Moreover, as any panoramic radiograph taken will have permanent tooth buds, the term mixed dentition was used instead of deciduous dentition. Table 1 mentions the inclusion and exclusion criteria for the selection of the dataset.

**Table 1:** Inclusion and exclusion criteria for the selection of the dataset

Inclusion criteria	Exclusion criteria
Panoramic radiograph showing a full set of permanent dentition	Panoramic radiograph with artifacts generated due to any technical or positioning errors or ghost images
Panoramic radiograph showing mixed dentition	Panoramic radiograph showing oral pathology which causes displacement/ missing/ resorption of teeth
	Panoramic radiograph with implants or crowns or bridges
	Panoramic radiograph with complete or partial anodontia
	Low-quality panoramic radiographs

Methodology

The dataset was divided into train, test, and valid sets [7] in the ratio 70:15:15 (as shown in Table 2). The training set is used to train the machine learning model, while the validation set is for fine-tuning it, and testing is to check the final model's performance.

**Table 2:** Division of datasets into train, test, and valid sets D: Deciduous; P: permanent

Data				
	Train	Valid	Test	Total
D	220	47	47	314
P	216	45	45	306
Total	436	92	92	620

The various steps used are listed and described below.

Data Pre-processing

**Figure 1:**
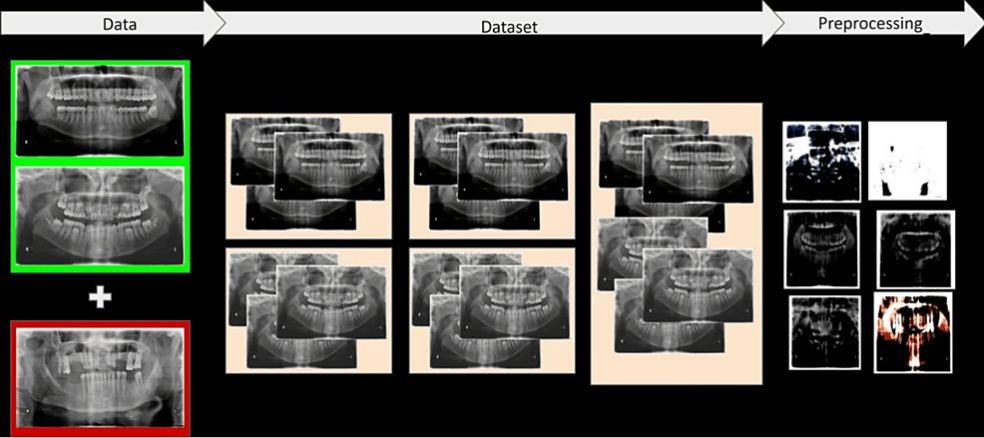
Data pre-processing steps

Data pre-processing was specific to the convolutional neural network (CNN) models. All the images were resized to 224*224, as the required input size of each model was 224*224. Keras library was used for the pre-processing of images. The pre-processing steps are shown in Figure 1.

Image resizing: Resizing the images to a uniform size ensures that the CNN models can extract features from the images in a consistent manner. This is important for improving the accuracy of the models.

Normalization: Normalization is a technique for scaling the pixel values of the images to a common range. In this case, the pixel values were normalized to lie between 0 and 1. This helps to improve the training process of the CNN models by making the data more consistent.

Data augmentation: Data augmentation is a technique for artificially increasing the size of the training dataset. This is done by applying transformations to the existing images, such as random flipping, cropping, and shearing. Data augmentation helps to prevent over-fitting by making the models more robust to variations in the input data.

Model Classification

**Figure 2:**
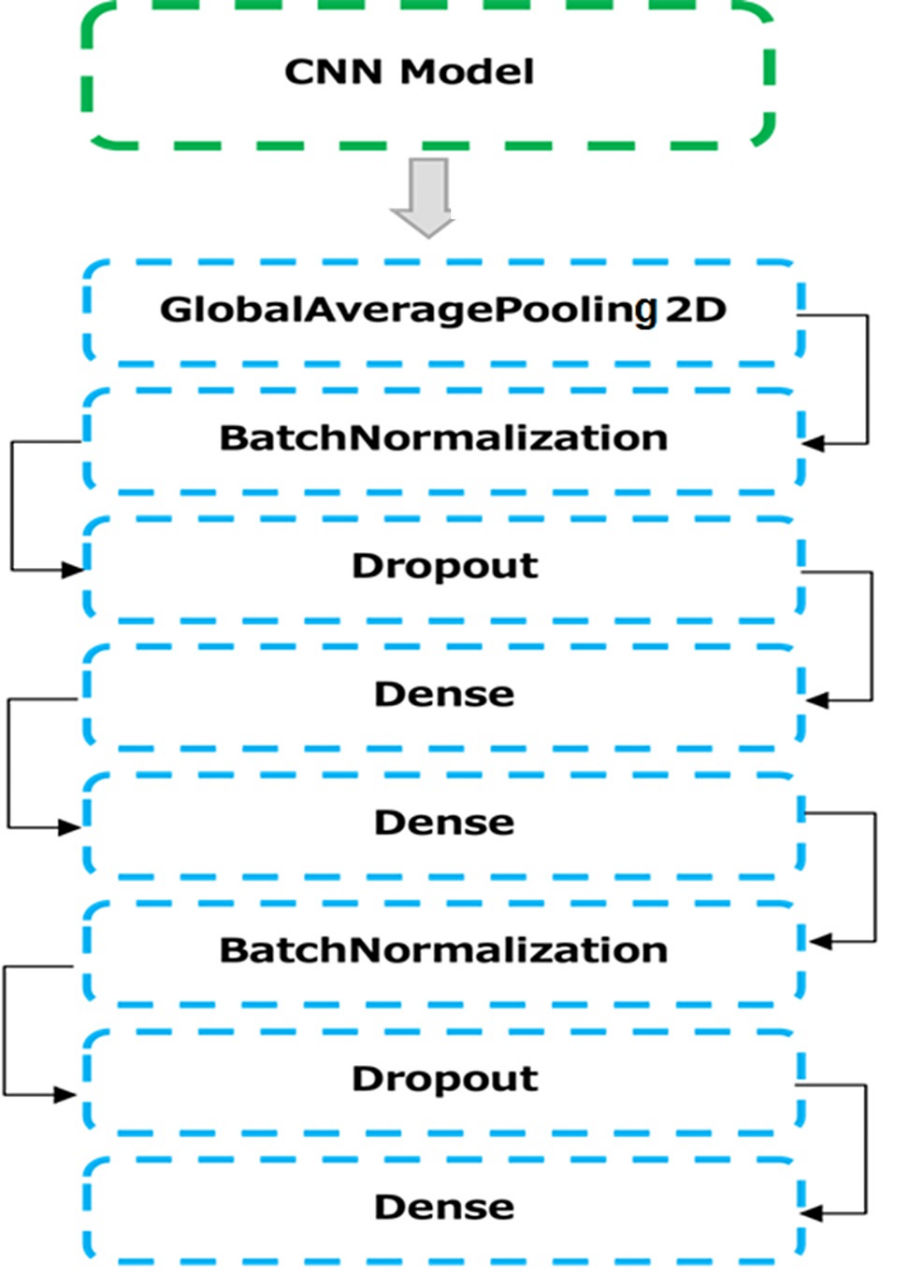
Addition of extra global average pooling, batch normalization, dropout, and dense layers in the CNN models CNN: Convolutional neural network

Transfer learning is a technique for reusing a pre-trained model on a new task. In this case, the researchers used pre-trained CNN models that had been trained on a large dataset of images. The pre-trained models were able to extract features from the images that were relevant to the task of deciduous tooth detection. These models have been stacked with additional layers as shown in Figure 2. These layers have been explained below.

Global average pooling: Global average pooling is a technique for reducing the spatial dimensions of the feature maps to a single vector. This simplifies the network's output and can help to improve the performance of the models.

Batch normalization: Batch normalization is a technique for normalizing the activations of layers. This can help to accelerate training and improve generalization.

Dropout: Dropout is a technique for randomly dropping neurons during training. This can help to prevent over-fitting and improve the robustness of the models.

Dense layer: The dense layer outputs the classification probability for the presence of deciduous teeth.

Apart from implementing the above-explained layers, the following techniques were used to prevent our model from overfitting:

Early stopping: Early stopping is a technique for halting the training process when the validation loss stops improving. The validation loss is the loss function evaluated on a separate validation dataset that is not used for training. By tracking the minimum value of validation loss, the researchers could identify the point where the model's performance on new data peaked. A patience level of 3 was chosen for general model convergence.

Learning rate scheduling: Learning rate scheduling is a technique for adjusting the learning rate during training. The learning rate is the parameter that controls how much the weights of the model are updated during each iteration of training. By adjusting the learning rate, the researchers could control the speed and direction of the training process. A patience level of 2 is specifically applied to learning rate adaptation. Further, when no improvement in performance was detected after certain epochs, the learning rate experienced a reduction by a factor of 0.3 in subsequent iterations.

**Figure 3:**
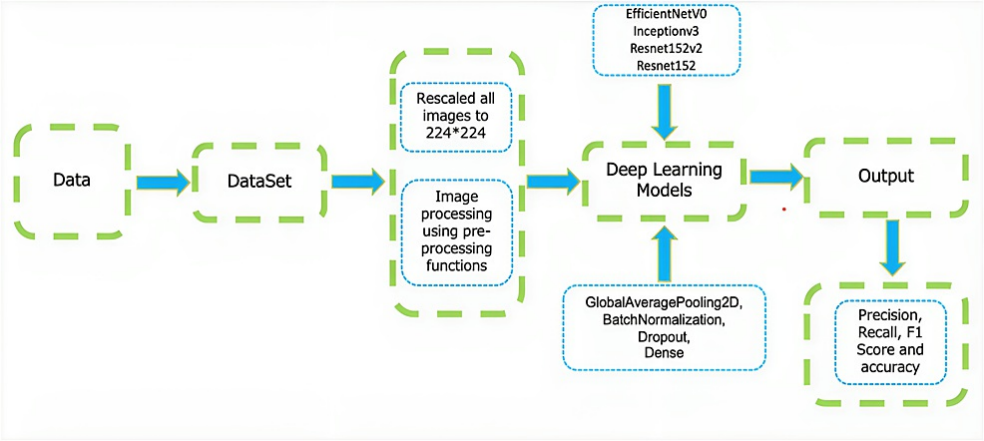
Complete end-to-end methodologies used for classification

An overview of the end-to-end methodology that has been followed and implemented is shown in Figure 3.

Keras library was used for data pre-processing and model training. Pre-trained CNN models were obtained from established deep-learning frameworks. Due to having a clean dataset with optimized pre-processing steps applied beforehand, our models converged relatively quickly within approximately 8-10 epochs before reaching this optimal point.

## Results

**Figure 4:**
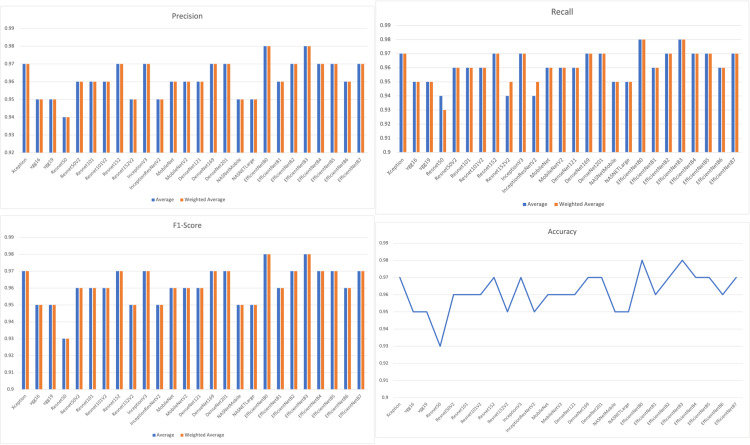
Results of various models (a) Precision, (b) recall, (c) F1-score, and (d) accuracy

The implementation results of eight types of deep learning models are listed and compared in Table 3. In our case, precision, recall, F1 score, and accuracy were calculated for permanent and deciduous teeth individually. Also, the results have been graphically depicted in Figure 4.

**Table 3:** Result comparison of various models used for classification

Algorithms	Precision		Recall		F1-Score		Accuracy
	Average	Weighted Average	Average	Weighted Average	Average	Weighted Average	
Xception	0.97	0.97	0.97	0.97	0.97	0.97	0.97
vgg16	0.95	0.95	0.95	0.95	0.95	0.95	0.95
vgg19	0.95	0.95	0.95	0.95	0.95	0.95	0.95
Resnet50	0.94	0.94	0.94	0.93	0.93	0.93	0.93
Resnet50V2	0.96	0.96	0.96	0.96	0.96	0.96	0.96
Resnet101	0.96	0.96	0.96	0.96	0.96	0.96	0.96
Resnet101V2	0.96	0.96	0.96	0.96	0.96	0.96	0.96
Resnet152	0.97	0.97	0.97	0.97	0.97	0.97	0.97
Resnet152V2	0.95	0.95	0.94	0.95	0.95	0.95	0.95
InceptionV3	0.97	0.97	0.97	0.97	0.97	0.97	0.97
InceptionResNetV2	0.95	0.95	0.94	0.95	0.95	0.95	0.95
MobileNet	0.96	0.96	0.96	0.96	0.96	0.96	0.96
MobileNetV2	0.96	0.96	0.96	0.96	0.96	0.96	0.96
DenseNet121	0.96	0.96	0.96	0.96	0.96	0.96	0.96
DenseNet169	0.97	0.97	0.97	0.97	0.97	0.97	0.97
DenseNet201	0.97	0.97	0.97	0.97	0.97	0.97	0.97
NASNetMobile	0.95	0.95	0.95	0.95	0.95	0.95	0.95
NASNETLarge	0.95	0.95	0.95	0.95	0.95	0.95	0.95
EfficientNetB0	0.98	0.98	0.98	0.98	0.98	0.98	0.98
EfficientNetB1	0.96	0.96	0.96	0.96	0.96	0.96	0.96
EfficientNetB2	0.97	0.97	0.97	0.97	0.97	0.97	0.97
EfficientNetB3	0.98	0.98	0.98	0.98	0.98	0.98	0.98
EfficientNetB4	0.97	0.97	0.97	0.97	0.97	0.97	0.97
EfficientNetB5	0.97	0.97	0.97	0.97	0.97	0.97	0.97
EfficientNetB6	0.96	0.96	0.96	0.96	0.96	0.96	0.96
EfficientNetB7	0.97	0.97	0.97	0.97	0.97	0.97	0.97

From the results shown in Table 3 and Figure 4, we can see that the EfficientNetB0 and EfficientNetB3 models outperform the other proposed models. The EfficientNetB0 and EfficientNet B3 models achieved 98% accuracy, 98% precision (positive predictions), 98% recall (sensitivity), and 98% F1 scores.

## Discussion

The rising adaptation of AI in healthcare has captured the attention of numerous researchers keen to contribute their expertise within this domain. In our present study, we endeavor to employ digital panoramic images together with various deep learning models for accurate classification and differentiation between deciduous and permanent teeth. To achieve this objective, a dataset consisting of 620 images was utilized, wherein several CNN-based transfer learning-based models were employed.

The application of transfer learning in image classification, particularly for medical images, has been proven to yield highly effective results. These models were utilized both as feature extractors and classification modules. We only fine-tuned and adjusted the last fully connected layers on our dataset [8]. Employing this approach enabled us to significantly reduce computational cost and time requirements without compromising the quality of outcomes.

Additionally, we fine-tuned various hyperparameters to achieve optimal performance. These included parameters such as learning rate, batch size, number of epochs, the dropout rate, activation function selection for neural networks' layers configuration, and tuning loss function that quantifies the error between predicted values and true labels during the model optimization process. Lastly, suitable optimization algorithms were chosen considering the subsequent attributes.

VGG, ResNet, Inception, Xception, MobileNet, DenseNet, NasNet, and EfficientNet are all popular deep learning models that have been widely used in various computer vision tasks. These models have significantly contributed to the advancement of image recognition and object detection. By incorporating different architectural designs and techniques such as depth-wise separable convolutions or network pruning strategies like skip connections or residual blocks, each model brings its unique approach to solving complex visual problems.

The VGG model is known for its simplicity with a stack of convolutional layers followed by fully connected layers. The only downside to this architecture is that it requires a significant amount of time for training. On the other hand, ResNet introduced residual connections which allow easier training of very deep networks while preserving information gradient flow.

Inception brought attention to factorizing convolutions into multiple parallel branches allowing better utilization of computing resources within network architecture. Building upon this concept, Xception further enhances efficiency without compromising performance by implementing separable convolutions at each layer.

MobileNets were designed specifically for mobile devices with low computational power. They utilize lightweight operations such as depth-wise separable convolutions resulting in faster inference times on resource-constrained platforms.

DenseNets propose densely connecting every previous layer with subsequent ones promoting feature reuse throughout the entire network architecture which boosts accuracy particularly when data availability is limited. However, this can lead to excessive connections between layers, resulting in reduced computational efficiency.

On the other hand, NasNet and EfficientNet are newer models that focus on automatic exploration for the most suitable network structure through neural architecture search, resulting in highly efficient and effective CNN models.

Each model brings its unique architectural design and techniques, allowing for better utilization of computing resources, faster inference times, and improved accuracy. These models have proven to be effective in solving complex visual problems and have been widely adopted in various applications and hence were selected for the present study.

After conducting an inter-group comparison, it was evident that the EfficientNetB0 and EfficientNetB3 models displayed superior performance when compared to the other proposed models. These two models consistently outperformed in various key parameters such as accuracy, precision, recall, and F1 score.

However, the performance of ResNet50 was significantly lower in terms of accuracy, recall, and F1score. This can be explained by its use of fewer layer blocks compared to ResNet101. Whereas ResNet50 employs a lesser number of three-layer blocks resulting in compromised accuracy.

A critical analysis was also conducted on relevant literature regarding similar research works. Table 4 provides a comprehensive comparison of these studies. However, it is worth noting that none of the aforementioned research specifically addresses the identification of deciduous teeth on panoramic images. Our study highlights substantial achievements in accurately distinguishing between deciduous and permanent teeth through classification techniques.

**Table 4:** A comparative analysis of related work in the field of dental radiography DL: Deep learning; ML: machine learning; CNN: convolutional neural network; VGG: visual geometry group; SVM: support vector machine

Author/year	Objective	Technique	Models used	Results
Imangaliyev S/2016[9]	To detect dental plaque	DL	Various CNN models	0.75
Oktay AB/2017[10]	Study placement of incisors, premolars, and molars	DL	Modified AlexNet	0.94
Lee JH/2018[11]	Detection of and diagnosis of dental caries	DL	GoogLeNet, InceptionV3	0.89
Bouchahma M/2019 [12]	Study tooth decay	DL	CNN	0.86
Sukegawa S/2020 [13]	Classification of 11 implant brands	DL	5 CNN models: a basic 6-layer CNN, VGG16, fine-tuned VGG16, VGG19, and fine-tuned VGG19	0.89
Takahashi T/2020 [14]	To identify dental implants	DL	YoloV3	0.85
Muresan MP/2020 [15]	Classification of panoramic dental X-rays into 14 classes	DL	Efficient Residual Factorized Convolutional Network	0.89
Verma D/2020 [16]	Classification of panoramic dental X-rays into 2 classes	ML & DL	SVM, Vanilla CNN, and a hybrid of SVM and Vanilla CNN	0.98
Cejudo JE /2021[17]	Classification of dental X-rays into panoramic, bitewing, periapical, and cephalometric	DL	ResNet34 and Capsule Network model	0.98
Caliskan S/ 2021 [18]	Detection and classification of submerged deciduous teeth	DL	Faster R-CNN	0.94

Based on the data presented in Tables 3, 4, EfficientNetB0 and EfficientNetB3 exhibited superior performance compared to other models. The better performance of EfficientNetB3 over other models can be attributed to the compound scaling method used to perform a grid search to find the relationship between different scaling dimensions of the baseline network under a fixed resource constraint.

From the results, it can be inferred that by utilizing AI models, a comparable or even higher level of expertise can be attained when it comes to identifying deciduous teeth in digital panoramic images.

One of the limitations of this study was the constrained dataset size available for testing and validation purposes.

For future experiments, we propose to apply the block-wise fine-tuning strategy to other deep learning models like GoogleNet to demonstrate the results of the current study and to build more robust classifiers. Furthermore, we also plan to create a model, which will be capable enough to identify the number of deciduous teeth as well as their stages of calcification and maturation.

## Conclusions

The results obtained in the current study provide a comparison of the efficiency of eight types of deep learning models in identifying the presence of deciduous teeth in digital panoramic images. Advanced machine learning models, namely EfficientNetB0 and EfficientNet B3, outperformed other models and were found to be more efficient. This research carries significant implications for pediatric dentists as it allows efficient screening of large datasets pertaining to deciduous as well as mixed dentition while enhancing diagnostic capabilities to identify dentition-related anomalies. Additionally, it provides additional benefits of helping in training residents and reducing variability among examiners by providing a standardized approach for identification purposes. As there are many reported variations in the eruption sequence of teeth among different ethnic groups, development of efficient AI systems can prove useful as an adjunct to human resources in large-sample research studies.
